# Real-world Evaluation of the Effectiveness of Sinopharm COVID-19 Vaccine Against Symptomatic COVID-19 in an Omicron-Dominant Setting in Mozambique: A Test-Negative, Case-Control Study

**DOI:** 10.1093/cid/ciaf093

**Published:** 2025-07-22

**Authors:** Igor Ubisse Capitine, Asma Binte Aziz, Alvaro Manhiça, Inês Tivane, Absalão Zumba, Milton Nhumba, Zamith Rebocho, Elias Miquicene, Neusa Nguenha, José Langa, Harshvardhan Shrivastava, Yunkai Yang, Shiyu Wang, Ju Yeon Park, Seung Eun Kyung, Young Ae You, Hyoryoung Lee, Eun Lyeong Park, Raphaël Rakotozandrindrainy, Sue Kyoung Jo, Chloe Sherliker, Jonathan D Sugimoto, Hyon Jin Jeon, Tabea Binger, Mohamadou Siribie, Deok Ryun Kim, Ilesh V Jani, Florian Marks, Birkneh Tilahun Tadesse

**Affiliations:** Instituto Nacional de Saúde, Maputo, Mozambique; International Vaccine Institute, Seoul, Republic of Korea; Instituto Nacional de Saúde, Maputo, Mozambique; Sofala Provincial Delegation, Instituto Nacional de Saúde, Mozambique; Sofala Provincial Delegation, Instituto Nacional de Saúde, Mozambique; Sofala Provincial Delegation, Instituto Nacional de Saúde, Mozambique; Sofala Provincial Delegation, Instituto Nacional de Saúde, Mozambique; Sofala Provincial Delegation, Instituto Nacional de Saúde, Mozambique; Sofala Provincial Delegation, Instituto Nacional de Saúde, Mozambique; Instituto Nacional de Saúde, Maputo, Mozambique; International Vaccine Institute, Seoul, Republic of Korea; China National Biotec Group Company Limited, China; China National Biotec Group Company Limited, China; International Vaccine Institute, Seoul, Republic of Korea; International Vaccine Institute, Seoul, Republic of Korea; International Vaccine Institute, Seoul, Republic of Korea; International Vaccine Institute, Seoul, Republic of Korea; International Vaccine Institute, Seoul, Republic of Korea; Madagascar Institute for Vaccine Research, University of Antananarivo, Antananarivo, Madagascar; International Vaccine Institute, Seoul, Republic of Korea; International Vaccine Institute, Seoul, Republic of Korea; International Vaccine Institute, Seoul, Republic of Korea; Department of Epidemiology, University of Washington, Seattle, Washington, USA; International Vaccine Institute, Seoul, Republic of Korea; Madagascar Institute for Vaccine Research, University of Antananarivo, Antananarivo, Madagascar; Department of Medicine, University of Cambridge, Cambridge, United Kingdom; International Vaccine Institute, Seoul, Republic of Korea; International Vaccine Institute, Seoul, Republic of Korea; Biomedical Research Unit, Burkina Institute of Global Health (BIGH), Ouagadougou, Burkina Faso; International Vaccine Institute, Seoul, Republic of Korea; Instituto Nacional de Saúde, Maputo, Mozambique; International Vaccine Institute, Seoul, Republic of Korea; Madagascar Institute for Vaccine Research, University of Antananarivo, Antananarivo, Madagascar; Department of Medicine, University of Cambridge, Cambridge, United Kingdom; The Hong Kong Jockey Club Global Health Institute, Hong Kong Special Administrative Region, China; Heidelberg Institute of Global Health, University of Heidelberg, Heidelberg, Germany; International Vaccine Institute, Seoul, Republic of Korea; Heidelberg Institute of Global Health, University of Heidelberg, Heidelberg, Germany

**Keywords:** COVID-19 vaccination, Sinopharm, effectiveness, Mozambique, test-negative design (TND)

## Abstract

**Background:**

We evaluated the effectiveness of the Sinopharm coronavirus disease 2019 (COVID-19) vaccine, introduced in Mozambique, against the Omicron variant of severe acute respiratory syndrome coronavirus 2 (SARS-CoV-2), prevalent from March 2022 to December 2023.

**Methods:**

A test-negative case-control study was nested in a community-based enhanced COVID-19 surveillance in seven healthcare facilities including mobile testing stations in the Dondo District, Mozambique, between March 2022 and December 2023. Participants were individuals aged ≥2 years with COVID-19–like symptoms for <10 days. Cases were those with polymerase chain reaction (PCR)–confirmed COVID-19. For each case, three PCR test–negative controls were matched, according to age (±5 years), sex, and date of PCR test (±7 days). Follow-up for all cases was conducted until disease resolution. Vaccine protection was assessed according to the association between complete vaccination and SARS-CoV-2 disease onset ≥14 days after vaccination.

**Results:**

The study did not reach the targeted sample size, and only a third were analyzed. A total of 253 cases were matched to 759 test-negative controls by age, sex, and testing date, a process known as *matching*. Among cases, 41% had one dose of Sinopharm, 53% had two doses, and 6% were unvaccinated. Among the test-negative controls, 37% had 1 dose, 57% had two doses, and 7% were unvaccinated. The adjusted vaccine effectiveness, calculated using matching and adjusted for age and PCR test date, was 18.0% (95% confidence interval, −85.3 to 63.7; *P* = .63). No COVID-19–positive participants required hospitalization.

**Conclusions:**

In an Omicron-dominant setting, two doses of the Sinopharm vaccine did not show significant protection against symptomatic COVID-19. However, as our analysis was based on data from only a third of enrolled individuals with confirmed vaccination status, these findings should be interpreted with caution. Our results underscore the importance of real-time vaccine effectiveness evaluations to inform optimal rollout strategies in low- and middle-income countries.

In 2020, the world faced the unprecedented challenge of the coronavirus disease 2019 (COVID-19) pandemic, caused by the severe acute respiratory syndrome coronavirus 2 (SARS-CoV-2) virus [1, 2]. By December 2023, Mozambique reported 233 731 confirmed COVID-19 cases and 2250 deaths [3]. Collaborative efforts led to the swift development of vaccines [4]. Phase 3 randomized-controlled trials demonstrated efficacy rates of 50%–95% against symptomatic disease [5, 6], thus, prompting the emergency use of several vaccines [7, 8].

The World Health Organization (WHO) endorsed the inactivated BBIBP-CorV (Sinopharm) vaccine, manufactured by the Beijing Institute of Biological Products in China, according to extensive evidence from a large multicountry phase 3 trial demonstrating 79% efficacy against symptomatic infection and hospitalization [9, 10]. Mozambique achieved one of the highest COVID-19 vaccination coverage rates in Africa by prioritizing use of safe and effective vaccines, coordinated planning, data-driven strategies, and effective communication [11]. This success was supported by a phased vaccination campaign using the Sinopharm vaccine under the COVAX framework [12].

However, whereas real-world evaluations of COVID-19 vaccines, such as those from Pfizer, Moderna, and Oxford AstraZeneca, have been conducted in developed countries, similar assessments for the Sinopharm vaccine are scarce in low- and middle-income countries, except Bangladesh and Morocco [13, 14]. To address this gap, we conducted a study using a prospective cohort follow-up and a nested test-negative design (TND) after a mass vaccination campaign with the Sinopharm vaccine, with an aim of evaluating the effectiveness of two doses of the Sinopharm vaccine in preventing reverse-transcription (RT) polymerase chain reaction (PCR)–confirmed symptomatic SARS-CoV-2 infection.

## METHODS

The study was conducted in the Dondo District and adjacent areas in Sofala Province, Mozambique. The study cohort was established after a mass vaccination campaign conducted by the Ministry of Health in Mozambique from 5 January to 12 March 2022, during which 54 975 residents received a first dose of the Sinopharm vaccine and 39 317 received a second dose. An additional 5895 individuals who missed the first dose were also vaccinated during the second phase. A baseline census was conducted immediately after the vaccination campaign, and a follow-up census was performed one year later, to estimate population movement and support vaccination status ascertainment efforts.

The censused population from April to June 2022 formed the basis of a cohort that was prospectively followed for COVID-19 via enhanced facility-based surveillance techniques. The surveillance was implemented in 7 of the 13 primary healthcare facilities in Dondo, including Canhandula, Dondo, Baptista, Mandril, Samora Machel, Mafambisse, and Mutua, between April 2022 and December 2023. These healthcare facilities were selected according to their experience in COVID-19 screening and testing, a well-defined catchment area, considerable population density, and adequate access. In addition, three community testing centers were set up in the surveillance catchment areas from which a considerable portion of cases originated during the study. All of these procedures were implemented to ensure consistent, broad, and equitable healthcare-seeking opportunities and testing access within the cohort.

We prospectively enrolled participants with COVID-19–like symptoms and conducted real-time RT-PCR testing. Cases were defined as individuals with positive test results; controls were those with negative results. Before initiation, the study protocol, informed consent form, and other essential study-specific documents were approved by the International Vaccine Institute’s institutional review board and Comité Nacional de Bioética para Saúde, Mozambique. The study was implemented according to the principles of the Declaration of Helsinki, International Conference on Harmonisation Good Clinical Practice guidelines, and Mozambique's ethical requirements.

### Enhanced Surveillance and Nested TND

Information sessions were conducted at the selected healthcare facilities and communities before study initiation. Individuals ≥2 years of age who were residents of the study area at the time of mass immunization, had not received a previous diagnosis of COVID-19 in the prior 90 days, and presented symptoms <10 days before testing, were eligible for enrollment in the nested TND study.

Eligible individuals at the outpatient departments of the selected health facilities or community surveillance posts were approached for enrollment if they exhibited symptoms consistent with COVID-19–like illness (eg, fever, cough, tiredness, loss of taste or smell, sore throat, headache, body aches, diarrhea, conjunctivitis, skin rash, or difficulty breathing). After obtaining written informed consent from adult participants and assent from minors, along with consent from their guardians, the participants’ sociodemographic data, history of illness, physical examination findings, and severity assessments were recorded in REDCap using a tablet, in compliance with ethical guidelines and local regulations.

Two nasopharyngeal swab samples were collected: one for rapid antigen testing and the other for RT-PCR testing. If the rapid antigen test result was positive, appropriate treatment was provided according to national guidelines. The nasopharyngeal swab samples for RT-PCR were stored in a cold box and sent to the Sofala Central Public Health Laboratory. The laboratory received the samples on the same day, and RT-PCR was performed the next day; results were available within 24–72 hours after sample receipt. Thus, the eligibility and clinical data were collected with blinding as to whether each patient was a test-positive case or a test-negative control, given that the PCR results were not available until the following day.

### Laboratory Procedure for Real-time RT-PCR and Genetic Sequencing

The nasopharyngeal swab samples were placed in a sterile tube containing 3 mL of viral transport medium (Wuxi Nest Biotechnology) and sent at 2°C–8°C to the Sofala Public Health laboratory on the same day of collection and stored at 2°C–8°C for up to 24 hours at the laboratory before testing. Next, 200 μL from each sample was used for viral RNA extraction with the QIAamp viral RNA kit (Qiagen), following the manufacturer's instructions. RT-PCR amplification was performed using the 2019-nCoV real-time fluorescent RT-PCR kit (BGI). Then 10 μL of RNA was added to 20 μL of the PCR mixture, giving a total reaction volume of 30 μL, as indicated by the manufacturer. The 2019-nCoV ORF1ab was the target region for amplification. RT was performed at 50°C for 20 minutes, an initial complementary DNA denaturation step at 95˚C for 10 minutes, and 40 cycles at 95°C for 15 minutes and 60°C for 30 seconds. A sample was declared positive for 2019-nCoV when the cycle threshold value of the targeted ORF1ab region was ≤38.

RNA was extracted from nasopharyngeal swab samples using the Qiagen Mini Kit 250 (Qiagen), according to the manufacturer's protocol. To generate complementary DNA and amplicons to be measured with real-time RT-PCR, the Abbott RealTime SARS-CoV-2 amplification kit (Abbott) was used, following the manufacturer's instructions. This is a dual-target assay for the RdRp and N genes. Only samples with a cycle threshold <30 were included for whole-genome sequencing. Next-generation sequencing was performed using the Illumina MiSeq and ISeq 100 platforms (Illumina). The COVIDseq library prep kit, COVIDseq CD indices, and v3 cartridge kits (500 cycles) were used for both platforms. Libraries were normalized to ensure that all samples were equally represented in the sequencing process and were then pooled into a single sequencing run that ended by loading the product onto the flow cell ready for sequencing. The sequencing machine outputs (Fastqs files) were analyzed using the Bioterra tool for bioinformatics analysis.

### Selection of Cases and Controls

In the cohort with enhanced COVID-19 surveillance, a TND was implemented to evaluate vaccine effectiveness (VE). In this design, individuals with RT-PCR test positivity for SARS-CoV-2 were defined as cases. Cases were selected on the basis of the onset of COVID-19–like symptoms and were excluded from further selection, as either case or controls, after their initial designation. Individuals presenting with COVID-19–like symptoms who tested negative for SARS-CoV-2 were defined as test-negative controls and were matched to cases in a 1:3 ratio according to three criteria: age on the testing date (±5 years), sex, and testing date (±7 days). To ensure unbiased analysis, all decisions regarding the eligibility of cases and controls were made without knowledge of vaccination status after the completion of the surveillance study. An independent statistician at the International Vaccine Institute, who was not involved in the study, generated the matched test-negative control set for analysis after database locking, to prevent observer bias.

### Follow-up in Symptomatic Individuals With RT-PCR Positivity for SARS-CoV-2

The study staff notified participants of positive results by telephone or through home visits. Participants with confirmed RT-PCR positivity for COVID-19 were followed up until symptom resolution, during four follow-up visits over as many as six weeks, if needed. If the symptoms resolved before six weeks, the follow-up ended, and the outcome was recorded. Participants without telephones were instructed to visit the health facility for follow-up. If contact was missed, the surveillance team made ≥3 attempts, including home visits, to collect information regarding severity and outcomes. All of the SARS-CoV-2–positive participants were monitored for any hospitalizations during the follow-up period.

### Vaccination Status Ascertainment

Vaccination data were collected during the baseline census in the REDCap database covering the entire Dondo District. These data were later linked to surveillance database through participants’ individual identifiers. Among the eligible census population, 85% had vaccination cards; for those without vaccination cards, data were cross-referenced against the national District Health Information Software 2 (DHIS2) database for accuracy. Vaccination status was reassessed during the follow-up census according to vaccination cards, together with data in the national DHIS2 database.

### Statistical Analysis

Initially, the required sample size for the evaluation of VE using the TND was calculated based on an assumed vaccine coverage of 30% among test-negative controls, an estimated VE of 70% (odds ratio [OR], 0.3), a type I error rate of 0.05, and an allocation ratio of 1:3 for cases to test-negative controls. Under these assumptions, the minimum number of cases needed to achieve 80% power was 412 (1236 controls). To account for the dominance of specific variants (70%) and potential dropouts (20%; 10% nonresponses and an additional 10% inflation), a total of 735 RT-PCR–confirmed case and 2205 test-negative controls were determined to be necessary for 80% power to detect VE by variant.

Baseline characteristics were analyzed to describe the study population and differences between cases and controls for TND analysis. For continuous variables, the means, SD, medians, and interquartile ranges were estimated; for categorical variables, counts with proportions were estimated. For continuous variables, *t*-tests were conducted; for categorical variables, χ^2^ or Fisher exact tests were used to provide a descriptive summary of differences in characteristics between test-positives and test-negatives. For comparison of matched cases and controls, Wald test from conditional logistic regression was used. Missing data for the occupation variable were excluded from the computation of the χ^2^  *P*-value, as this variable had some missing observations. However, all other baseline variables were complete and did not have any missing data. Since the occupation variable was not included in the VE analysis model, imputation was not performed.

Each case was matched to three controls according to the three matching criteria, age on the testing date (±5 years), sex, and testing date (±7 days). Two matching methods—matching and propensity score matching (PSM)—were applied by using these identified variables. The primary evaluation relied on the VE estimate, calculated with a 95% confidence interval (CI) through the exact matching method. As part of the sensitivity analysis, PSM was applied to compute VE. Eligible participants verified as either unvaccinated or vaccinated with the Sinopharm vaccine were considered potential candidates for PSM. Propensity scores were estimated using a logistic regression model, incorporating age, PCR testing date, and sex as predictors of RT-PCR results. Each case was matched with controls using an optimal matching method to minimize the total absolute difference in propensity scores. For sex, exact matching was applied.

Complete vaccination was defined as receipt of two doses of Sinopharm with ≥14 days having elapsed since the second vaccine dose. One-dose vaccination was defined as receipt of the first vaccine dose with >21 days having elapsed after the first dose. Incomplete two-dose vaccination was defined as receipt of two doses of the Sinopharm vaccine with <14 days having elapsed after the second dose. Individuals not receiving any COVID-19 vaccine were considered unvaccinated.

For the primary endpoint analysis, we used the conditional multivariable regression model to adjust for confounding variables. Because a range of ages (±5 years) and testing dates (±7 days) were used for matching, these variables were also included as covariates in the model to account for any potential residual confounding. Other potential confounders, including underlying conditions (presence of asthma, chronic kidney disease being treated with dialysis, chronic lung disease, diabetes, hemoglobin disorders, immunocompromise, liver disease, serious heart conditions, or severe obesity), behavioral characteristics (estimated time spent outside, mask-wearing habit outdoors/indoors, social/physical distancing, handwashing, or close contact with a person with known COVID-19), and prior diagnosis with COVID-19 were also considered as candidate covariates in the multivariate models. A stepwise selection method was applied to identify variables for inclusion, with a *P*-value threshold of .1 for entry and .3 for exit. Interaction terms between vaccination status and other covariates were also considered as candidate variables in the stepwise selection process; however, none were identified as significant.

The final VE estimates were calculated with the adjusted OR (aOR) for vaccination with the following formula: VE = (1 − aOR) × 100%. We quantified and measured the precision of any effect modification, including VE estimation by subgroup. Each analysis was conducted on the subset of matched cases and controls that met the definitions for “exposed” and “unexposed” for each strata-specific analysis. If effect modification existed, VE and CIs are reported for each subgroup separately. ORs were estimated by exponentiation of the coefficient for vaccination in the models, and standard errors for these coefficients were used to calculate two-sided *P*-values and 95% CIs for the ORs. Model diagnostics included evaluations of goodness of fit, identification of outliers, and assessment of multicollinearity. All statistical analyses were performed using SAS software, version 9.4, and R software, version 4.4.0.

## RESULTS

### Assembly of the Study Population

**Figure 1.**
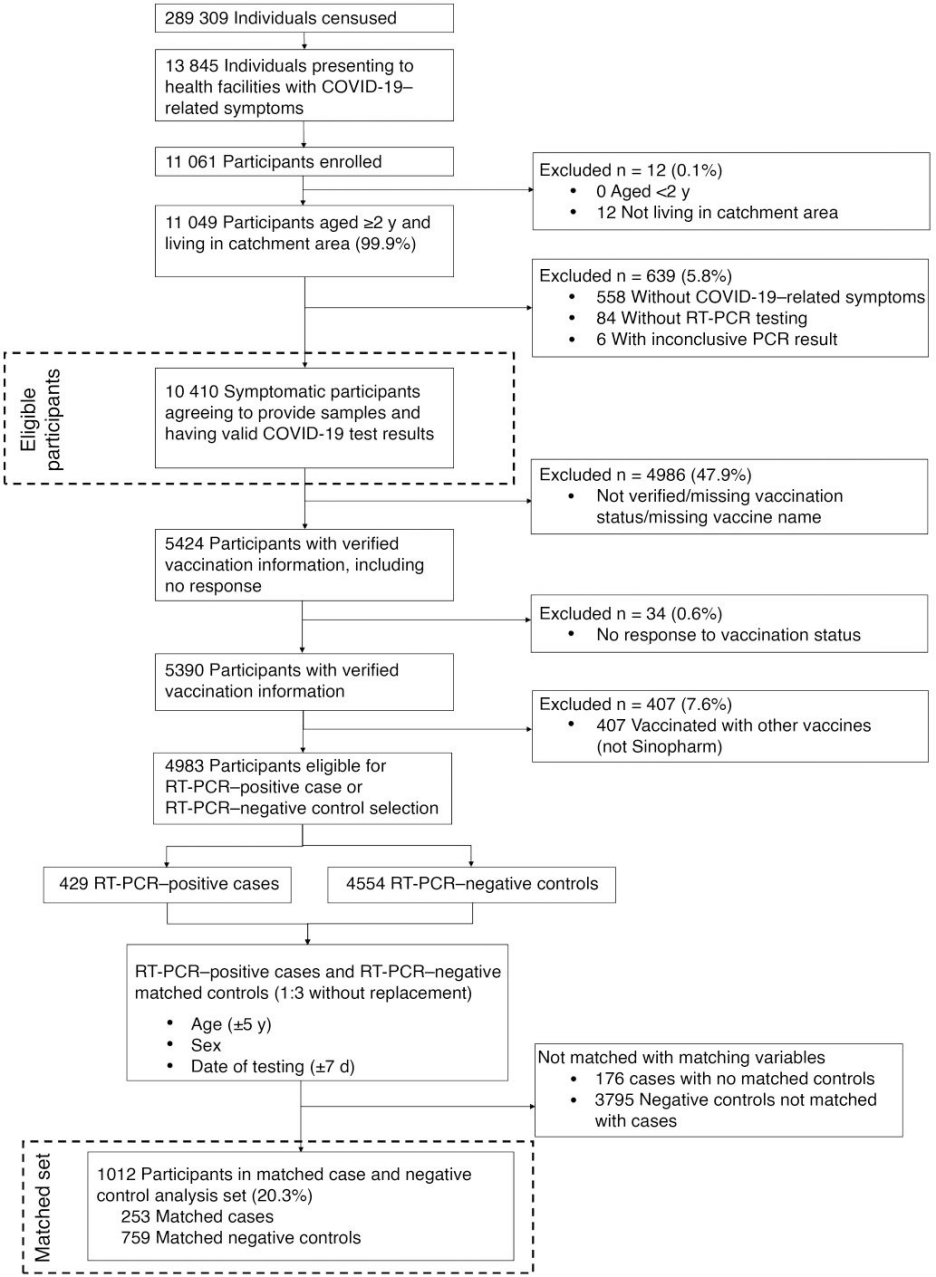
Flow diagram of participant disposition. Abbreviations: COVID-19, coronavirus disease 2019; RT-PCR, reverse-transcription polymerase chain reaction.

From March 2022 to December 2023, a total of 13 845 patients at the healthcare facilities who presented with COVID-19­–like symptoms were approached to participate, 75% (10 410/13 845) of whom were enrolled. Among them, a total of 48% (4983/10 410) had verified vaccination information and were eligible for the study after exclusion of 407 who received vaccines other than Sinopharm. A total of 429 RT-PCR–positive cases and 4554 potential negative controls were identified. On the basis of predefined matching criteria, 176 cases and 3795 controls were unmatched (Figure 1). Ultimately, 253 cases and 759 test-negative controls, on the basis of matching, were included in the final analysis (Figure 1). Whole-genome sequencing revealed that all sequenced RT-PCR–positive cases were infected with the Omicron variant.

A comparison of characteristics through descriptive statistics between the cases and test-negative controls, among eligible participants and within the matched set, indicated comparable baseline characteristics between groups. Most matched patients (70% [712/1012]) were female and 2–45 years of age (Table 1). The most frequently reported COVID-19 symptoms were headache (89% [906/1012]), followed by cough (85% [865/1012]), muscle/body pain (58% [585/1012]), and sore throat (31% [310/1012]) among the matched group **(**Supplementary Table 1**)**. Immunocompromised status was the most common underlying condition among eligible participants reporting any underlying condition (42% [209/492]) and the matched set of cases and controls (40% [18/45]) and was similar among groups (Supplementary Table 1). Symptom severity by case and control groups is summarized in Supplementary Table 1. Among the participants, six individuals experienced severe symptoms (one case and five controls). None required hospitalization, and all were managed by study physicians without complications. All six individuals with severe symptoms had received the Sinopharm vaccine. The case was vaccinated with two complete doses. Among the five controls, three had received two complete doses of Sinopharm, while the remaining two had received one dose.

**Table 1.** Sociodemographic Characteristics of Eligible Participants and Members of Matched Set

Characteristic	Eligible Participants, No. (%)^a^				Members of Matched Set, No. (%)^a^			
	Test Positive (n = 705)	Test-Negative (n = 9705)	*P*-Value^b^	Total (n = 10 410)	Cases (n = 253)	Negative Controls (n = 759)	*P*-Value^c^	Total (n = 1012)
Sex								
Male	280 (39.7)	4217 (43.5)	.053	4497 (43.2)	75 (29.6)	225 (29.6)	NA	300 (29.6)
Female	425 (60.3)	5488 (56.5)		5913 (56.8)	178 (70.4)	534 (70.4)		712 (70.4)
Age								
Mean (SD), y	32.9 (15.9)	32.5 (16.0)	.52	32.5 (16.0)	32.1 (12.3)	32.1 (12.3)	.72	32.1 (12.3)
Median, y	29.0	29.0		29.0	28.0	29.0		28.0
Range, y	2.0–93.0	2.0–100.0		2.0–100.0	18.0–72.0	13.0–75.0		13.0–75.0
Age group								
2–45 y	563 (79.9)	7781 (80.2)	.8400	8344 (80.2)	216 (85.4)	652 (85.9)	.55	868 (85.8)
≥46 y	142 (20.1)	1924 (19.8)		2066 (19.8)	37 (14.6)	107 (14.1)		144 (14.2)
Range of RT-PCR dates	1 Jun 2022 to 26 Dec 2023	2 Mar 2022 to 26 Dec 2023		2 Mar 2022 to 26 Dec 2023	18 Jun 2022 to 22 Dec 2023	16 Jun 2022 to 26 Dec 2023		16 Jun 2022 to 26 Dec 2023
RT-PCR date by period								
Mar–Jun 2022	16 (2.3)	451 (4.6)	.003^d^	467 (4.5)	7 (2.8)	18 (2.4)	.55	25 (2.5)
Jul–Dec 2022	221 (31.3)	3124 (32.2)	.64	3345 (32.1)	125 (49.4)	351 (46.2)	.02^d^	476 (47.0)
Jan–Jun 2023	247 (35.0)	2888 (29.8)	.003^d^	3135 (30.1)	65 (25.7)	221 (29.1)	.008^d^	286 (28.3)
Jul–Dec 2023	221 (31.3)	3242 (33.4)	.26	3463 (33.3)	56 (22.1)	169 (22.3)	.78	225 (22.2)
Occupation^e^								
Housewife	81 (18.1)	887 (20.1)	.30	968 (19.9)	41 (21.4)	153 (28.6)	.03^d^	194 (26.7)
Pensioners/retired	11 (2.5)	66 (1.5)	.12	77 (1.6)	3 (1.6)	6 (1.1)	.52	9 (1.2)
Household helping hand	18 (4.0)	180 (4.1)	.95	198 (4.1)	8 (4.2)	22 (4.1)	.85	30 (4.1)
Farmer/fisherman/forester	98 (21.9)	878 (19.9)	.32	976 (20.1)	49 (25.5)	121 (22.6)	.15	170 (23.4)
Tailor/barber/craftsman	2 (0.4)	22 (0.5)	>.99	24 (0.5)	2 (1.0)	1 (0.2)	.14	3 (0.4)
Traders/business owner	38 (8.5)	304 (6.9)	.21	342 (7.0)	20 (10.4)	38 (7.1)	.15	58 (8.0)
Public administration/government	20 (4.5)	191 (4.3)	.90	211 (4.3)	7 (3.6)	15 (2.8)	.49	22 (3.0)
Private company	26 (5.8)	332 (7.5)	.18	358 (7.4)	10 (5.2)	27 (5.0)	.92	37 (5.1)
Nonprofit institution	3 (0.7)	41 (0.9)	.79	44 (0.9)	1 (0.5)	6 (1.1)	.98	7 (1.0)
Student	4 (0.9)	44 (1.0)	>.99	48 (1.0)	3 (1.6)	3 (0.6)	.17	6 (0.8)
Self-employed	3 (0.7)	26 (0.6)	.75	29 (0.6)	1 (0.5)	3 (0.6)	.83	4 (0.6)
Unemployed	68 (15.2)	695 (15.8)	.74	763 (15.7)	30 (15.6)	83 (15.5)	.77	113 (15.5)
Other	42 (9.4)	478 (10.8)	.34	520 (10.7)	13 (6.8)	46 (8.6)	.26	59 (8.1)
Don't know	4 (0.9)	23 (0.5)	.31	27 (0.6)	2 (1.0)	4 (0.7)	.98	6 (0.8)
No response	1 (0.2)	16 (0.4)	>.99	17 (0.4)	0 (0.0)	2 (0.4)	.98	2 (0.3)
Not applicable	29 (6.5)	225 (5.1)	.22	254 (5.2)	2 (1.0)	5 (0.9)	.86	7 (1.0)
Missing	257	5297	…	5554	61	224	…	285

Abbreviations: NA, Not Applicable; RT-PCR, reverse-transcription polymerase chain reaction.

^a^Data represent no. (%) of participants unless otherwise specified.

^b^ *P*-values calculated using χ^2^ or Fisher exact test.

^c^ *P*-values calculated from conditional logistic regression using Wald test.

^d^Significant at *P* < .05.

^e^For occupations, percentages are computed excluding participants with missing occupations (n = 448 and n = 4408 for test-positive and test-negative group, respectively; n = 192 and n = 535 for case and negative control groups, respectively).

### Vaccination Status Among Eligible Participants and the Matched Set

Among 5390 eligible participants with vaccination information, 33% (1792/5390) received one dose of Sinopharm, 53% (2871/5390) received two doses, and 6% (319/5390) were unvaccinated. This distribution was similar among the RT-PCR–positive cases (1 dose, 35% [160/460]; 2 doses, 52% [239/460]; and unvaccinated, 7% [30/460]) and RT-PCR–negative controls (33% [1632/4930], 53% [2632/4930], and 6% [289/4930], respectively). In 253 cases and 759 exactly matched controls, the level of vaccination was similarly distributed: 41% (103/253) of cases and 37% (279/759) of controls had received one dose, while 53% (135/253) of cases and 57% (430/759) of controls had received two doses. Approximately 6% of participants in both groups (15/253 cases and 50/759 controls) were unvaccinated (Table 2).

**Table 2.** Vaccination Status of the Eligible Participants and Members of the Matched Set

Vaccination Status	Eligible Participants With Vaccination Information, No. (%)				Members of Matched Set, No. (%)			
	Test-Positive (n = 462)	Test-Negative (n = 4962)	*P*-Value^a^	Total (n = 5424)	Cases (n = 253)	Negative Controls (n = 759)	*P*-Value^b^	Total (n = 1012)
Sinopharm								
1 Dose	160 (34.6)	1632 (32.9)	.45	1792 (33.0)	103 (40.7)	279 (36.8)	.23	382 (37.7)
2 Doses	239 (51.7)	2632 (53.0)	.59	2871 (52.9)	135 (53.4)	430 (56.7)	.32	565 (55.8)
3 Doses	0 (0.0)	1 (0.0)	>.99	1 (0.0)	0 (0.0)	0 (0.0)	NA	0 (0.0)
Not vaccinated	30 (6.5)	289 (5.8)	.56	319 (5.9)	15 (5.9)	50 (6.6)	.71	65 (6.4)
No response	2 (0.4)	32 (0.6)	>.99	34 (0.6)	…	…	…	…
Other vaccine								
1 Dose	17 (3.7)	247 (5.0)	.21	264 (4.9)	…	…	…	…
2 Doses	14 (3.0)	129 (2.6)	.58	143 (2.6)	…	…	…	…
Heterologous vaccine	5 (35.7)	24 (18.6)	.16	29 (20.3)	…	…	…	…
Homologous vaccine	9 (64.3)	105 (81.4)	.16	114 (79.7)	…	…	…	…

^a^ *P*-values calculated using χ^2^ or Fisher exact tests.

^b^ *P*-values calculated from conditional logistic regression using Wald test.

### VE Against RT-PCR–Confirmed COVID-19 Disease, With a TND

**Figure 2.**
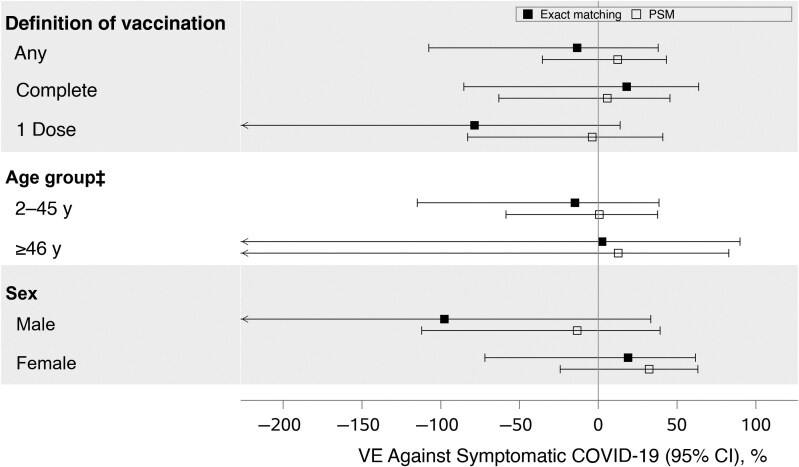
Adjusted vaccine effectiveness (VE) against reverse-transcription polymerase chain reaction (RT-PCR)–confirmed coronavirus disease 2019 (COVID-19) disease, with 95% confidence intervals (CIs). Complete vaccination was defined as ≥14 days after receipt of two completed doses of the Sinopharm vaccine; one dose, as >21 days after one dose of the Sinopharm vaccine. Patients with either the first or second dose date missing were included only in the overall analysis as vaccinated patients, and patients were included in the analysis only if at least one case and one control remained in each matched set. The age group is determined based on the age of the matched cases. Vaccination status has been modified based on the dates of vaccine administration and of the RT-PCR test. For matching, VE is adjusted for age and the date of RT-PCR test; for propensity score matching (PSM), VE is adjusted for age, date of RT-PCR test, social/physical distancing behavior, and prior COVID-19 diagnosis. Matching variables age and date of RT-PCR date were included by default. Other covariates were selected using stepwise variable selection.

To evaluate the VE of two complete doses of the Sinopharm vaccine against RT-PCR­–confirmed COVID-19, we analyzed 317 Sinopharm-vaccinated patients, including 105 RT-PCR–positive cases. The crude VE was 15.9% (95% CI, −89.6 to 62.7; *P* = .68), which increased to 18.0% (−85.3 to 63.7; *P* = .63) after adjustment for age and PCR test date. For those who received only one dose, the crude VE was −75.7% (95% CI, −262.7 to 14.9), with an adjusted VE of −78.6% (−270.1 to 13.8). The overall crude VE after ≥1 dose was −12.1% (95% CI, −104.4 to 38.5; *P* = .71). After adjustment for confounders (age and date of PCR testing), the VE was −13.5% (95% CI, −107.6 to 37.9; *P* = .68) (Table 3 and Figure 2).

**Table 3.** Vaccine Effectiveness Against RT-PCR–Confirmed COVID-19 by Definition of Vaccination, Age Group, and Sex, Using a Test-Negative Design

Variable	Cases, No.			Matched Controls, No.			Crude VE (95% CI), %	*P*-Value	Adjusted VE^a^ (95% CI), %	*P*-Value
	Total	Vaccinated	Unvaccinated	Total	Vaccinated	Unvaccinated				
Definition of vaccination^b^										
Complete	119	105	14	231	212	19	15.9 (−89.6 to 62.7)	.68	18.0 (−85.3 to 63.7)^c^	.63
1 Dose	97	85	12	176	143	33	−75.7 (−262.7 to 14.9)	.13	−78.6 (−270.1 to 13.8)	.12
Any	253	238	15	759	709	50	−12.1 (−104.4 to 38.5)	.71	−13.5 (−107.6 to 37.9)	.68
Age group^d^										
2–45 y	216	202	14	648	601	47	−13.1 (−110.8 to 39.3)	.70	−14.9 (−114.9 to 38.6)	.66
≥46 y	37	36	1	111	108	3	0.0 (−861.4 to 89.6)	>.99	2.3 (−853.6 to 90.0)	.98
Sex										
Male	75	71	4	225	202	23	−97.1 (−480.6 to 33.1)	.22	−97.6 (−485.4 to 33.3)	.22
Female	178	167	11	534	507	27	20.2 (−68.4 to 62.2)	.55	18.8 (−72.0 to 61.7)	.59

Abbreviations: CI, confidence interval; VE, vaccine effectiveness.

^a^Adjusted for age and date of the reverse-transcription polymerase chain reaction (RT-PCR) test.

^b^Vaccination status modified based on the dates of vaccine administration and of the RT-PCR test. Complete vaccination was defined as ≥14 days after receipt of two completed doses of the Sinopharm vaccine; one dose, as >21 days after one dose of the Sinopharm vaccine. Patients with either the first or second dose date missing were included only in the overall analysis as vaccinated patients, and patients were included in the analysis only if at least 1 case and 1 control remained in each matched set.

^c^Primary end-point analysis.

^d^Age group based on the age group of the matched case.

In a sensitivity analysis using PSM in a larger cohort of 622 patients (191 cases), we observed similar trends to those in the matching analysis after complete vaccination. The crude VE was 2.5 (95% CI, −66.8 to 43.0; *P* = .93) after complete vaccination. After adjustment for confounders (age, date of RT-PCR test, social/physical distancing behavior, and prior COVID-19 diagnosis), the VE was 5.6% (95% CI, −63.1 to 45.4; *P* = .84) (Table 4 and Figure 2).

**Table 4.** Vaccine Effectiveness Against RT-PCR–Confirmed COVID-19 by Definition of Vaccination, Age Group, and Sex, Using Propensity Score Matching

Variable	Cases, No.			Matched Controls, No.			Crude VE (95% CI), %	*P*-Value	Adjusted VE^a^ (95% CI), %	*P*-Value
	Total	Vaccinated	Unvaccinated	Total	Vaccinated	Unvaccinated				
Definition of vaccination^b^										
Complete	217	191	26	405	364	41	2.5 (−66.8 to 43.0)	.93	5.6 (−63.1 to 45.4)^c^	.84
1 Dose	153	130	23	259	218	41	−2.7 (−79.4 to 41.2)	.92	−3.9 (−83.0 to 41.0)	.89
Any	429	399	30	1287	1207	80	11.7 (−35.9 to 42.6)	.57	12.2 (−35.4 to 43.1)	.56
Age group^d^										
2–45 y	330	303	27	990	918	72	11.6 (−39.1 to 43.9)	.59	0.6 (−58.5 to 37.7)	.98
≥46 y	99	96	3	297	289	8	11.8 (−247.8 to 77.6)	.86	12.6 (−345.0 to 82.8)	.87
Sex										
Male	158	144	14	474	427	47	−12.9 (−109.8 to 39.2)	.70	−13.5 (−112.1 to 39.3)	.69
Female	271	255	16	813	780	33	31.6 (−24.8 to 62.6)	.22	32.3 (−24.2 to 63.1)	.21

Abbreviations: CI, confidence interval; VE, vaccine effectiveness.

^a^Adjusted for age, date of reverse-transcription polymerase chain reaction (RT-PCR) test, social/physical distancing behavior, and prior coronavirus disease 2019 diagnosis; matching variables (age and date of RT-PCR test) were included by default. Other covariates were selected using stepwise variable selection.

^b^Vaccination status modified based on the dates of vaccine administration and of the RT-PCR test. Complete vaccination was defined as ≥14 days after receipt of two completed doses of the Sinopharm vaccine; one dose, as >21 days after one dose of the Sinopharm vaccine. Patients with either the first or second dose date missing were included only in the overall analysis as vaccinated patients, and patients were included in the analysis only if at least one case and one control remained in each matched set.

^c^Primary end-point analysis.

^d^Age group based on the age group of the matched case.

## DISCUSSION

To our knowledge, this study is the first real-world evaluation of the effectiveness of the Sinopharm vaccine in Mozambique, as well as one of the few studies reporting real-world evidence after COVID-19 vaccine introduction in sub-Saharan Africa. The absence of electronic health record systems in low- and middle-income countries made real-world evidence extremely difficult to generate during COVID-19 vaccination rollout in these settings.

In this real-world evaluation of the effectiveness of the Sinopharm COVID-19 vaccine administered through a government campaign, we observed no significant protection against nonsevere clinical forms of COVID-19. The adjusted VE, assessed with matching, was 18.0%, which went even lower with PSM, to 5.6%. Despite these findings, it is important to note that none of the cases in our study required hospitalization or resulted in death, suggesting that the vaccine likely provided substantial protection against severe outcomes. This aligns with the growing understanding that COVID-19 vaccines, including Sinopharm, may have a limited impact on preventing mild disease, but can reduce disease severity [15].

Our findings contribute to the global evidence base on COVID-19 vaccines, addressing a critical gap in understanding their effectiveness in resource-limited settings. Similar studies have reported mixed results for Sinopharm. For instance, two studies in Morocco have assessed the effectiveness of the Sinopharm vaccine administered as a primary series. The first study reported an 89% effectiveness against COVID-19 hospitalizations across all age groups, but this finding was specific to the Alpha strain [13]. The second study demonstrated that a Sinopharm booster dose effectively increased protection against death and severe hospitalization associated with the Omicron variant [16]. Conversely, data from Bangladesh indicated no protection against symptomatic Delta-related COVID-19 cases 19 weeks after vaccination [14]. These disparities highlight the complex interplay between vaccine performance, variant evolution, and population-specific factors.

Several factors might explain the low Sinopharm VE in our study. Waning immunity likely played a role, as the average time from the last vaccine dose to COVID-19 diagnosis in our study population was approximately 10 months. Evidence from messenger RNA–based and other vaccine platforms suggests that protection against symptomatic infection diminishes significantly within 8–11 months after the primary series, particularly against immune-evasive variants, such as Omicron [17, 18]. This reduction in VE over time, combined with the ability of the Omicron variant to evade immunity, likely contributed to the lower VE observed in our study. These findings underscore the importance of variant-specific evaluations and the development of updated COVID-19 vaccines tailored to emerging strains [19, 20].

Our study used a TND to minimize biases related to healthcare-seeking behavior and differential access to testing [21]. However, this approach has inherent limitations that merit consideration. To address potential selection bias, we implemented enhanced surveillance within a defined catchment area, incorporating diverse healthcare facilities and community testing centers. This strategy promoted equitable access to testing and minimized geographic and socioeconomic disparities. In addition, by including symptomatic individuals irrespective of their vaccination status, we reduced the likelihood of differential healthcare-seeking behavior affecting case or control classification. To mitigate the risk of disease misclassification, we relied on RT-PCR testing, the gold standard for COVID-19 diagnosis, which minimizes the likelihood of false-negatives among test-negative controls. The analysis focused exclusively on individuals presenting with COVID-19–like symptoms, enhancing the likelihood that test-negative individuals were true-negatives.

All participants with positive RT-PCR results were followed up prospectively to accurately capture their disease resolution status, minimizing the risk of reclassification errors due to subsequent symptom development. Vaccination status was meticulously documented using vaccination cards, census data, and the national DHIS2 database. Blinded data collection procedures ensured that vaccination status was recorded independently of case/control designation, as the statistician responsible for data processing was unaware of participants' vaccination status. This multi-tiered approach minimized misclassification risk and ensured comprehensive data capture. To further reduce confounding, cases and controls were matched by age, sex, and test date. Matching on test date (±7 days) also controlled for seasonal variations, changes in disease incidence, and circulating variants during the study period, which spanned multiple seasons (April 2022–December 2023) in an Omicron-dominant setting.

Despite these strengths, our findings should be interpreted with caution due to several limitations. Tracking and linking individuals' vaccination status to health outcomes was challenging but essential for evaluating the vaccination program's effectiveness. Natural disasters, such as cyclones, disrupted surveillance activities critical for monitoring disease spread and vaccine effects. Consequently, only 18% of eligible participants with validated vaccination status were included in the matched case and control groups, as many did not meet predefined matching criteria. This limitation may have introduced selection bias if there were differences between eligible participants and those included in the analysis. However, among both eligible participants and those in the matched set, 93% were vaccinated, with >50% receiving two doses of Sinopharm, and their baseline characteristics were comparable.

In addition, pandemic-related lockdowns and travel restrictions affected on-site monitoring and quality control activities. Nevertheless, data quality was maintained through subsequent on-site visits once restrictions were lifted. The inability to evaluate VE against severe outcomes limits the generalizability of our findings to nonsevere cases. Furthermore, while the enhanced surveillance cohort provided comprehensive data, results may not be fully generalizable to asymptomatic infections or populations with different healthcare-seeking behaviors. Including diverse healthcare facilities and community testing centers helped minimize biases related to geographic and socioeconomic disparities.

Although the sample size was smaller than originally estimated, it provided valuable insights into clinically meaningful differences in VE, particularly in the context of real-world data collection. The dynamic nature of the pandemic made achieving the desired sample size challenging. While the wide CIs reflect inherent variability, this does not diminish the importance of our findings in offering an initial estimate of VE under real-world conditions. However, caution is warranted when interpreting VE estimates by variant due to reduced power. While the study design focused on symptomatic individuals who sought care at testing facilities, this may have led to an underestimation of VE, as individuals with milder symptoms or asymptomatic infections may not have presented for testing. This is an inherent limitation of the TND [22], as individuals not seeking medical care are not captured, potentially biasing VE estimates toward lower effectiveness. Thus, these findings should be interpreted cautiously, recognizing that they may not fully represent VE across the spectrum of COVID-19 severity, including patients with asymptomatic or mild COVID-19 who did not seek care.

In conclusion, in this real-world evaluation of the Sinopharm COVID-19 vaccine in Mozambique, we observed no significant protection against non-severe symptomatic COVID-19. However, the absence of severe outcomes, such as hospitalizations or deaths, suggests that the Sinopharm COVID-19 vaccine likely provided protection against severe disease. These findings underscore the critical need for ongoing real-world evaluations of COVID-19 vaccines in resource-limited settings to address population-specific challenges, evolving variants, and gaps in baseline immunity. Although our study provides valuable insights, the wide CIs and lack of statistical significance in the VE estimates mean that these results should be interpreted with caution. Further research with larger sample sizes and more precise estimates is needed to better understand the effectiveness of COVID-19 vaccines, particularly in resource-limited settings.

## Supplementary Material

ciaf093_Supplementary_Data

## Supplementary Data

Supplementary materials are available at *Clinical Infectious Diseases* online. Consisting of data provided by the authors to benefit the reader, the posted materials are not copyedited and are the sole responsibility of the authors, so questions or comments should be addressed to the corresponding author.

## Notes

***Author contributions.*** A. B. A. wrote the first draft. Data analysis was done by J. Y. P., S. E. K., Y. A. Y., H. L., E.L.P., and D. R. K. All authors contributed to data interpretation and manuscript review, led by I. V. J., F. M., and B. T. T. All authors had access to the analyzed datasets.

***Acknowledgments.*** The authors are grateful to all participants in the study and to their external service providers (laboratories and clinical suppliers) for their invaluable assistance in performing the study. They are grateful to Paul Oloo, Saurabh Ahuja, and Dorota Zdankiewicz from Coalition for Epidemic Preparedness Innovations for the oversight. Special acknowledgment to Nílsio Sabonete, Sifrónia Massunganhe, Edi Fulai, Maria Maviga, Unícia Chibale, Falume Chale, and João Luís Manuel for their significant contributions to the study.

***Disclaimer.*** The funding organization had no role in the design, collection, analysis, or interpretation of data, or the writing of the manuscript. China National Biotec Group Company Limited had no role in the design, collection and analysis. The findings and conclusions in this report are those of the authors and do not necessarily represent the official position of the International Vaccine Institute, the Instituto Nacional de Saúde, the University of Antananarivo, Madagascar Institute for Vaccine Research, or any of the collaborating partners in this study.

***Financial support.*** This work was supported by the Coalition for Epidemic Preparedness Innovations (grant PRJ-6447).

***Supplement sponsorship.*** This article appears as part of the supplement “COVID-19 Vaccine Research in Africa,” sponsored by the International Vaccine Institute and funded by the Coalition for Epidemic Preparedness Innovations (CEPI).
