# Supplementary material for: Real-world Evaluation of the Effectiveness of Sinopharm COVID-19 Vaccine Against Symptomatic COVID-19 in an Omicron-Dominant Setting in Mozambique: A Test-Negative, Case-Control Study
Source: Clin Infect Dis. 2025 Jul 22;80(Suppl 1):S47–56. doi: 10.1093/cid/ciaf093 (PMC12282512; doi:10.1093/cid/ciaf093)
Supplement: ciaf093_Supplementary_Data [file ciaf093_supplementary_data.docx]

**Supplementary table 1: COVID-19-related symptoms/underlying condition**

|  | **Eligible Participant** | | | | **Matched set** | | | |
| --- | --- | --- | --- | --- | --- | --- | --- | --- |
|  | **Test Positive** | **Test Negative** | **p-value**^†^ | **Total** | **Cases** | **Negative Controls** | **p-value**^‡^ | **Total** |
|  | **N=705** | **N=9705** |  | **N=10410** | **N=253** | **N=759** |  | **N=1012** |
| Symptoms within the last 10 days, n (%) |  |  |  |  |  |  |  |  |
| Fever | 50 (7.1%) | 703 (7.2%) | 0.88 | 753 (7.2%) | 18 (7.1%) | 29 (3.8%) | **0.034** | 47 (4.6%) |
| Cough | 617 (87.5%) | 8461 (87.2%) | 0.80 | 9078 (87.2%) | 220 (87.0%) | 645 (85.0%) | 0.43 | 865 (85.5%) |
| Fatigue | 93 (13.2%) | 1446 (14.9%) | 0.22 | 1539 (14.8%) | 35 (13.8%) | 101 (13.3%) | 0.83 | 136 (13.4%) |
| Muscle/Body pain | 441 (62.6%) | 5671 (58.4%) | **0.032** | 6112 (58.7%) | 158 (62.5%) | 427 (56.3%) | 0.082 | 585 (57.8%) |
| Chills | 6 (0.9%) | 96 (1.0%) | 0.73 | 102 (1.0%) | 5 (2.0%) | 10 (1.3%) | 0.45 | 15 (1.5%) |
| Headache | 634 (89.9%) | 8669 (89.3%) | 0.62 | 9303 (89.4%) | 235 (92.9%) | 671 (88.4%) | **0.047** | 906 (89.5%) |
| Loss of smell | 24 (3.4%) | 313 (3.2%) | 0.80 | 337 (3.2%) | 11 (4.3%) | 23 (3.0%) | 0.29 | 34 (3.4%) |
| Loss of taste | 53 (7.5%) | 908 (9.4%) | 0.10 | 961 (9.2%) | 24 (9.5%) | 57 (7.5%) | 0.29 | 81 (8.0%) |
| Sore Throat | 218 (30.9%) | 2944 (30.3%) | 0.74 | 3162 (30.4%) | 88 (34.8%) | 222 (29.2%) | 0.095 | 310 (30.6%) |
| Nasal congestion | 153 (21.7%) | 1933 (19.9%) | 0.25 | 2086 (20.0%) | 60 (23.7%) | 154 (20.3%) | 0.25 | 214 (21.1%) |
| Runny nose | 112 (15.9%) | 935 (9.6%) | **<.0001** | 1047 (10.1%) | 32 (12.6%) | 56 (7.4%) | **0.0099** | 88 (8.7%) |
| Nausea or Vomiting | 21 (3.0%) | 375 (3.9%) | 0.24 | 396 (3.8%) | 7 (2.8%) | 21 (2.8%) | 1.0 | 28 (2.8%) |
| Diarrhea | 10 (1.4%) | 239 (2.5%) | 0.080 | 249 (2.4%) | 6 (2.4%) | 21 (2.8%) | 0.74 | 27 (2.7%) |
| Shortness of breath/Difficulty in breathing | 13 (1.8%) | 343 (3.5%) | **0.017** | 356 (3.4%) | 8 (3.2%) | 32 (4.2%) | 0.46 | 40 (4.0%) |
|  |  |  |  |  |  |  |  |  |
| Underlying condition, n (%) |  |  |  |  |  |  |  |  |
| Any underlying condition | 28 (4.0%) | 464 (4.8%) | 0.33 | 492 (4.7%) | 12 (4.7%) | 33 (4.3%) | 0.79 | 45 (4.4%) |
| Asthma* | 6 (21.4%) | 104 (22.4%) | 0.90 | 110 (22.4%) | 2 (16.7%) | 4 (12.1%) | NA | 6 (13.3%) |
| Chronic kidney disease being treated with dialysis* | 0 (0.0%) | 3 (0.6%) | 1.0 | 3 (0.6%) | 0 (0.0%) | 0 (0.0%) | NA | 0 (0.0%) |
| Chronic lung disease* | 0 (0.0%) | 4 (0.9%) | 1.0 | 4 (0.8%) | 0 (0.0%) | 0 (0.0%) | NA | 0 (0.0%) |
| Diabetes* | 0 (0.0%) | 7 (1.5%) | 1.0 | 7 (1.4%) | 0 (0.0%) | 0 (0.0%) | NA | 0 (0.0%) |
| Hemoglobin disorders* | 0 (0.0%) | 1 (0.2%) | 1.0 | 1 (0.2%) | 0 (0.0%) | 0 (0.0%) | NA | 0 (0.0%) |
| Immunocompromise* | 10 (35.7%) | 199 (42.9%) | 0.46 | 209 (42.5%) | 4 (33.3%) | 14 (42.4%) | NA | 18 (40.0%) |
| Liver disease* | 0 (0.0%) | 0 (0.0%) | NA | 0 (0.0%) | 0 (0.0%) | 0 (0.0%) | NA | 0 (0.0%) |
| Working at nursing homes/long-term care facilities* | 0 (0.0%) | 0 (0.0%) | NA | 0 (0.0%) | 0 (0.0%) | 0 (0.0%) | NA | 0 (0.0%) |
| Serious heart conditions* | 5 (17.9%) | 70 (15.1%) | 0.60 | 75 (15.2%) | 2 (16.7%) | 5 (15.2%) | NA | 7 (15.6%) |
| Severe obesity* | 1 (3.6%) | 3 (0.6%) | 0.21 | 4 (0.8%) | 1 (8.3%) | 0 (0.0%) | NA | 1 (2.2%) |
| Others* | 7 (25.0%) | 124 (26.7%) | 0.84 | 131 (26.6%) | 4 (33.3%) | 14 (42.4%) | NA | 18 (40.0%) |
|  |  |  |  |  |  |  |  |  |
| Severe symptoms at enrollment, n (%) |  |  |  |  |  |  |  |  |
| Oxygen saturation <90% on room air | 0 (0.0%) | 48 (0.5%) | 0.076 | 48 (0.5%) | 0 (0.0%) | 1 (0.1%) | 0.98 | 1 (0.1%) |
| High Respiratory rate | 1 (0.1%) | 10 (0.1%) | 0.54 | 11 (0.1%) | 0 (0.0%) | 0 (0.0%) | NA | 0 (0.0%) |
| Signs of severe respiratory distress | 1 (0.1%) | 11 (0.1%) | 0.57 | 12 (0.1%) | 1 (0.4%) | 0 (0.0%) | 0.98 | 1 (0.1%) |
| Signs requiring immediate life sustaining support | 2 (0.3%) | 50 (0.5%) | 0.58 | 52 (0.5%) | 0 (0.0%) | 4 (0.5%) | 0.98 | 4 (0.4%) |
|  |  |  |  |  |  |  |  |  |
| Diagnosed with COVID-19 greater than 90 days in the past, Yes n (%) | 9 (1.3%) | 51 (0.5%) | **0.019** | 60 (0.6%) | 5 (2.0%) | 7 (0.9%) | 0.19 | 12 (1.2%) |
|  |  |  |  |  |  |  |  |  |
| Symptomatic if Yes, n (%) | 9 (100.0%) | 47 (92.2%) | 1.0 | 56 (93.3%) | 5 (100.0%) | 7 (100.0%) | NA | 12 (100.0%) |

[Note] SD: Standard Deviation; N: Number of total participants; n: Number of participants who reported each demographic characteristics; %: Percentages (100*n/N)

* For each underlying conditions, N = Number of total participants who reported any underlying condition

† The p-value is calculated using Chi Squared test or Fisher's exact test;

‡ The p-value is calculated from conditional logistic regression using the Wald test.

**Supplementary table 2: COVID-19 related behaviour**

|  | **Eligible Participant** | | | | **Matched set** | | | |
| --- | --- | --- | --- | --- | --- | --- | --- | --- |
|  | **Test Positive** | **Test Negative** | **p-value** | **Total** | **Cases** | **Negative Controls** | **p-value**^‡^ | **Total** |
|  | **N=705** | **N=9705** |  | **N=10410** | **N=253** | **N=759** |  | **N=1012** |
| Estimated time of spending outside, n (%) |  |  |  |  |  |  |  |  |
| Less than 2 hours, 1 | 38 (5.4%) | 545 (5.6%) | 0.54* | 583 (5.6%) | 12 (4.7%) | 28 (3.7%) | 0.91 | 40 (4.0%) |
| 2-4 hours, 2 | 200 (28.4%) | 2564 (26.4%) |  | 2764 (26.6%) | 72 (28.5%) | 212 (27.9%) |  | 284 (28.1%) |
| 4-8 hours, 3 | 257 (36.5%) | 3829 (39.5%) |  | 4086 (39.3%) | 90 (35.6%) | 277 (36.5%) |  | 367 (36.3%) |
| 8-10 hours, 4 | 175 (24.8%) | 2339 (24.1%) |  | 2514 (24.1%) | 69 (27.3%) | 205 (27.0%) |  | 274 (27.1%) |
| More than 10 hours, 5 | 35 (5.0%) | 428 (4.4%) |  | 463 (4.4%) | 10 (4.0%) | 37 (4.9%) |  | 47 (4.6%) |
| Mean of score (SD) | 3.0 (1.0) | 3.0 (1.0) | 0.93^†^ | 3.0 (1.0) | 3.0 (1.0) | 3.0 (0.9) | 0.53 | 3.0 (0.9) |
| Mask wearing outdoors, n (%) |  |  |  |  |  |  |  |  |
| Always, 1 | 24 (3.4%) | 412 (4.2%) | **0.0012*** | 436 (4.2%) | 7 (2.8%) | 18 (2.4%) | 0.78 | 25 (2.5%) |
| Frequently, 2 | 139 (19.7%) | 1437 (14.8%) |  | 1576 (15.1%) | 52 (20.6%) | 131 (17.3%) |  | 183 (18.1%) |
| Sometimes, 3 | 325 (46.1%) | 4794 (49.4%) |  | 5119 (49.2%) | 118 (46.6%) | 377 (49.7%) |  | 495 (48.9%) |
| Infrequently, 4 | 175 (24.8%) | 2639 (27.2%) |  | 2814 (27.0%) | 62 (24.5%) | 185 (24.4%) |  | 247 (24.4%) |
| Never, 5 | 42 (6.0%) | 423 (4.4%) |  | 465 (4.5%) | 14 (5.5%) | 48 (6.3%) |  | 62 (6.1%) |
| Mean of score (SD) | 3.1 (0.9) | 3.1 (0.9) | 0.48^†^ | 3.1 (0.9) | 3.1 (0.9) | 3.2 (0.9) | 0.38 | 3.1 (0.9) |
| Mask wearing indoors, n (%) |  |  |  |  |  |  |  |  |
| Always, 1 | 2 (0.3%) | 26 (0.3%) | 0.13* | 28 (0.3%) | 1 (0.4%) | 2 (0.3%) | 0.48 | 3 (0.3%) |
| Frequently, 2 | 12 (1.7%) | 115 (1.2%) |  | 127 (1.2%) | 3 (1.2%) | 10 (1.3%) |  | 13 (1.3%) |
| Sometimes, 3 | 76 (10.8%) | 1178 (12.1%) |  | 1254 (12.0%) | 34 (13.4%) | 80 (10.5%) |  | 114 (11.3%) |
| Infrequently, 4 | 131 (18.6%) | 2110 (21.7%) |  | 2241 (21.5%) | 44 (17.4%) | 164 (21.6%) |  | 208 (20.6%) |
| Never, 5 | 484 (68.7%) | 6276 (64.7%) |  | 6760 (64.9%) | 171 (67.6%) | 503 (66.3%) |  | 674 (66.6%) |
| Mean of score (SD) | 4.5 (0.8) | 4.5 (0.8) | 0.16^†^ | 4.5 (0.8) | 4.5 (0.8) | 4.5 (0.8) | 0.75 | 4.5 (0.8) |
| Social/Physical distancing, n (%) |  |  |  |  |  |  |  |  |
| Always, 1 | 44 (6.2%) | 937 (9.7%) | **0.0043*** | 981 (9.4%) | 17 (6.7%) | 46 (6.1%) | 0.56 | 63 (6.2%) |
| Frequently, 2 | 64 (9.1%) | 1056 (10.9%) |  | 1120 (10.8%) | 21 (8.3%) | 75 (9.9%) |  | 96 (9.5%) |
| Sometimes, 3 | 327 (46.4%) | 4281 (44.1%) |  | 4608 (44.3%) | 140 (55.3%) | 384 (50.6%) |  | 524 (51.8%) |
| Infrequently, 4 | 253 (35.9%) | 3116 (32.1%) |  | 3369 (32.4%) | 72 (28.5%) | 237 (31.2%) |  | 309 (30.5%) |
| Never, 5 | 17 (2.4%) | 315 (3.2%) |  | 332 (3.2%) | 3 (1.2%) | 17 (2.2%) |  | 20 (2.0%) |
| Mean of score (SD) | 3.2 (0.9) | 3.1 (1.0) | **0.0018**^†^ | 3.1 (1.0) | 3.1 (0.8) | 3.1 (0.9) | 0.45 | 3.1 (0.8) |
| Handwashing, n (%) |  |  |  |  |  |  |  |  |
| Always, 1 | 84 (11.9%) | 1296 (13.4%) | **0.044*** | 1380 (13.3%) | 34 (13.4%) | 90 (11.9%) | 0.82 | 124 (12.3%) |
| Frequently, 2 | 268 (38.0%) | 4055 (41.8%) |  | 4323 (41.5%) | 98 (38.7%) | 318 (41.9%) |  | 416 (41.1%) |
| Sometimes, 3 | 187 (26.5%) | 2466 (25.4%) |  | 2653 (25.5%) | 78 (30.8%) | 224 (29.5%) |  | 302 (29.8%) |
| Infrequently, 4 | 159 (22.6%) | 1783 (18.4%) |  | 1942 (18.7%) | 41 (16.2%) | 124 (16.3%) |  | 165 (16.3%) |
| Never, 5 | 7 (1.0%) | 105 (1.1%) |  | 112 (1.1%) | 2 (0.8%) | 3 (0.4%) |  | 5 (0.5%) |
| Mean of score (SD) | 2.6 (1.0) | 2.5 (1.0) | **0.0051**^†^ | 2.5 (1.0) | 2.5 (0.9) | 2.5 (0.9) | 0.92 | 2.5 (0.9) |
|  |  |  |  |  |  |  |  |  |
| Travel Recently, Yes n (%) | 3 (0.4%) | 82 (0.8%) | 0.23* | 85 (0.8%) | 1 (0.4%) | 7 (0.9%) | 0.41 | 8 (0.8%) |
|  |  |  |  |  |  |  |  |  |
| Attend the social events/gathering, Yes n (%) | 26 (3.7%) | 349 (3.6%) | 0.90* | 375 (3.6%) | 16 (6.3%) | 41 (5.4%) | 0.58 | 57 (5.6%) |
|  |  |  |  |  |  |  |  |  |
| Close-contact with a known COVID-19 case in the last two weeks (14 days), n (%) |  |  |  |  |  |  |  |  |
| Yes | 3 (0.4%) | 8 (0.1%) | **0.034*** | 11 (0.1%) | 0 (0.0%) | 1 (0.1%) | 0.98 | 1 (0.1%) |
| No | 654 (92.8%) | 9049 (93.2%) | 0.63* | 9703 (93.2%) | 235 (92.9%) | 703 (92.6%) | 0.89 | 938 (92.7%) |
| Don’t know | 48 (6.8%) | 648 (6.7%) | 0.89* | 696 (6.7%) | 18 (7.1%) | 55 (7.2%) | 0.94 | 73 (7.2%) |
|  |  |  |  |  |  |  |  |  |
| Relationship if Yes, n (%) |  |  |  |  |  |  |  |  |
| Household member | 1 (33.3%) | 5 (62.5%) | 0.55* | 6 (54.5%) | - | 0 (0.0%) | NA | 0 (0.0%) |
| Non-co-habiting family member/friend | 1 (33.3%) | 0 (0.0%) | 0.27* | 1 (9.1%) | - | 0 (0.0%) | NA | 0 (0.0%) |
| Coworker | 0 (0.0%) | 0 (0.0%) | NA | 0 (0.0%) | - | 0 (0.0%) | NA | 0 (0.0%) |
| Schoolmate | 1 (33.3%) | 2 (25.0%) | 1.0* | 3 (27.3%) | - | 0 (0.0%) | NA | 0 (0.0%) |
| Client | 0 (0.0%) | 0 (0.0%) | NA | 0 (0.0%) | - | 0 (0.0%) | NA | 0 (0.0%) |
| Patient (if health worker) | 0 (0.0%) | 0 (0.0%) | NA | 0 (0.0%) | - | 0 (0.0%) | NA | 0 (0.0%) |
| Housemaid/caregiver | 0 (0.0%) | 0 (0.0%) | NA | 0 (0.0%) | - | 0 (0.0%) | NA | 0 (0.0%) |
| Other(s) | 0 (0.0%) | 1 (12.5%) | 1.0* | 1 (9.1%) | - | 1 (100.0%) | NA | 1 (100.0%) |

* The p-value is calculated using Chi Squared test or Fisher's exact test;

† The p-value is calculated using t-test comparing the means of score.

‡ The p-value is calculated from conditional logistic regression using the Wald test.
